# By more ways than one: Rapid convergence at hydrothermal vents shown by 3D anatomical reconstruction of *Gigantopelta* (Mollusca: Neomphalina)

**DOI:** 10.1186/s12862-017-0917-z

**Published:** 2017-03-01

**Authors:** Chong Chen, Katsuyuki Uematsu, Katrin Linse, Julia D. Sigwart

**Affiliations:** 1 Marine Laboratory, Queen’s University Belfast, 12-13 The Strand, Portaferry, Northern Ireland; 2 Department of Subsurface Geobiological Analysis and Research (D-SUGAR), Japan Agency for Marine-Earth Science and Technology (JAMSTEC), 2-15 Natsushima, Yokosuka, Kanagawa 237-0061 Japan; 3 Marine Works Japan Ltd., 3-54-1 Oppamahigashi, Yokosuka, 237-0063 Japan; 4 British Antarctic Survey, High Cross, Cambridge, UK; 5 Berkeley, Museum of Paleontology, University of California, VLSB 1101, Berkeley, CA 94720 USA

**Keywords:** Adaptation, Anatomy, Convergence, Evolution, Extreme environment, Symbiosis

## Abstract

**Background:**

Extreme environments prompt the evolution of characteristic adaptations. Yet questions remain about whether radiations in extreme environments originate from a single lineage that masters a key adaptive pathway, or if the same features can arise in parallel through convergence. Species endemic to deep-sea hydrothermal vents must accommodate high temperature and low pH. The most successful vent species share a constrained pathway to successful energy exploitation: hosting symbionts. The vent-endemic gastropod genus *Gigantopelta*, from the Southern and Indian Oceans, shares unusual features with a co-occurring peltospirid, the ‘scaly-foot gastropod’ *Chrysomallon squamiferum*. Both are unusually large for the clade and share other adaptive features such as a prominent enlarged trophosome-like oesophageal gland, not found in any other vent molluscs.

**Results:**

Transmission electron microscopy confirmed endosymbiont bacteria in the oesophageal gland of *Gigantopelta*, as also seen in *Chrysomallon*. They are the only known members of their phylum in vent ecosystems hosting internal endosymbionts; other vent molluscs host endosymbionts in or on their gills, or in the mantle cavity. A five-gene phylogenetic reconstruction demonstrated that *Gigantopelta* and *Chrysomallon* are not phylogenetically sister-taxa, despite their superficial similarity. Both genera have specialist adaptations to accommodate internalised endosymbionts, but with anatomical differences that indicate separate evolutionary origins. Hosting endosymbionts in an internal organ within the host means that all resources required by the bacteria must be supplied by the animal, rather than directly by the vent fluid. Unlike *Chrysomallon*, which has an enlarged oesophageal gland throughout post-settlement life, the oesophageal gland in *Gigantopelta* is proportionally much smaller in juveniles and the animals likely undergo a trophic shift during ontogeny. The circulatory system is hypertrophied in both but the overall size is smaller in *Gigantopelta.* In contrast with *Chrysomallon*, *Gigantopelta* possesses true ganglia and is gonochoristic.

**Conclusions:**

Key anatomical differences between *Gigantopelta* and *Chrysomallon* demonstrate these two genera acquired a similar way of life through independent and convergent adaptive pathways. What appear to be the holobiont’s adaptations to an extreme environment, are driven by optimising bacteria’s access to vent nutrients. By comparing *Gigantopelta* and *Chrysomallon*, we show that metazoans are capable of rapidly and repeatedly evolving equivalent anatomical adaptations and close-knit relationships with chemoautotrophic bacteria, achieving the same end-product through parallel evolutionary trajectories.

**Electronic supplementary material:**

The online version of this article (doi:10.1186/s12862-017-0917-z) contains supplementary material, which is available to authorized users.

## Background

All “extreme” environments prompt the evolution of characteristic anatomical and physiological features in organisms that exploit them. Animals inhabiting deep-sea hydrothermal vents face high pressure, strong gradients of pH and temperature, hydrogen sulfide, methane, and heavy metals [1, 2]. The foundation of these ecosystems is microbial chemosynthesis [3] and many endemic vent metazoans host symbiotic bacteria either on the body surface as epibonts (e.g., crustaceans *Shinkaia*, *Rimicaris*), or within their tissues as endosymbionts (e.g., siboglinid tubeworms *Riftia*, and molluscs *Calyptogena*, *Bathymodiolus*) [4]. Chemosynthesis generates energy through the oxidation of sulfide and methane, the key oxidant being oxygen in the surrounding water [5]. These animals usually present conspicuous physiological and anatomical adaptations to serve the endosymbiont; for example siboglinid tubeworms have a specialised organ, the trophosome, to house their sulfur-oxidising bacteria and have haemoglobin that binds both oxygen and sulfur to supply the bacteria with its two key resources [6]. Vent ecosystems fall into different faunal provinces, with most species geographically restricted to particular ridge systems [7–9]. Regardless of faunal composition, in all biogeographic regions the dominant vent metazoan species consistently have close relationships with chemosynthetic bacteria [7, 9]. The current understanding is that majority of vent endemic taxa have evolved relatively recently and all dominant taxa are highly modified for lifestyle specific to hydrothermal vents [10, 11]. Yet, hosting symbiotic bacteria is the unifying feature of vent-endemic primary consumers, and thus appears to be the singular constrained evolutionary pathway to successful exploitation of the energy from vents.

Vent ecosystems are characterised by local concentrations of biomass, matching that of tropical coral reefs [1], but in vents that biomass is dominated by a few species forming huge aggregations around fluid effluents [7]. In many cases, the dominant species are molluscs, which include at least seven phylogenetically independent origins of relationships with endosymbiotic bacteria [12–14]. Vents on the East Scotia Ridge (ESR), Southern Ocean, and the Southwest Indian Ridge (SWIR), for example, are dominated by large members of the vent-endemic gastropod family Peltospiridae [15], namely *Gigantopelta* spp. and the ‘scaly-foot gastropod’ *Chrysomallon squamiferum*, which both live in high density aggregations [9, 16, 17]. *Chrysomallon* is distinct for its dermal armature of scales that cover the outer surface of the foot, unique among living gastropods [18–20].

While *Gigantopelta* spp. lack scales, they share several features with *Chrysomallon* that make both gastropod genera unusual in context of their clade, such as a large body size (>45 mm, compared to typical sizes in other taxa of 10–15 mm [21]; meaning a 10–50 times increase in body volume) and an enlarged oesophageal gland [16]. Some gastropod limpets in other families found in reducing environments have enlarged oesophageal pouches [22, 23], but these species are not from hydrothermal vents, and the presence of symbionts within their oesophageal structures has never been directly observed except in the case of *Lepetella* [23]. Previous studies of *Chrysomallon* reported sulfur-oxidising ɣ-proteobacteria housed within the oesophageal gland [14, 24]. Stable isotope analyses using adult *G. chessoia* specimens demonstrated likely reliance on endosymbionts for nutrition [25, 26], but the existence of endosymbionts had not been directly observed. One aim of the present study was to confirm the presence of microbes in the oesophageal gland tissues in *Gigantopelta*.

*Chrysomallon* and *Gigantopelta* are the only vent animals, except siboglinid tubeworms, that house endosymbionts within an enclosed part of the body not in direct contact with vent fluid, an arrangement that implies all resources required by the bacteria must be supplied by the host animal [20]. In most vent taxa that host chemoautotrophic bacteria, including other molluscs such as *Alviniconcha* snails and *Calyptogena* clams, endosymbionts are contained within the gill (or mantle cavity, in the case of *Lurifax vitreus* [27]), where they can more or less directly access the chemical resources they require [28]. The same is true in symbiotrophic molluscs from other reducing ecosystems, ranging from intertidal muds to methane seeps [29, 30]. All siboglinid worms have a single evolutionary origin of their internal gut-based endosymbiosis [31], yet it is unclear whether the peltospirid gastropods *Chrysomallon* and *Gigantopelta* represent a single or separate acquisition of endosymbiosis.

Here, we present new evidence of key anatomical and phylogenetic differences between *Chrysomallon* and *Gigantopelta* that demonstrate these two genera acquired a similar way of life through two different adaptive pathways.

## Results

**Fig. 1:**
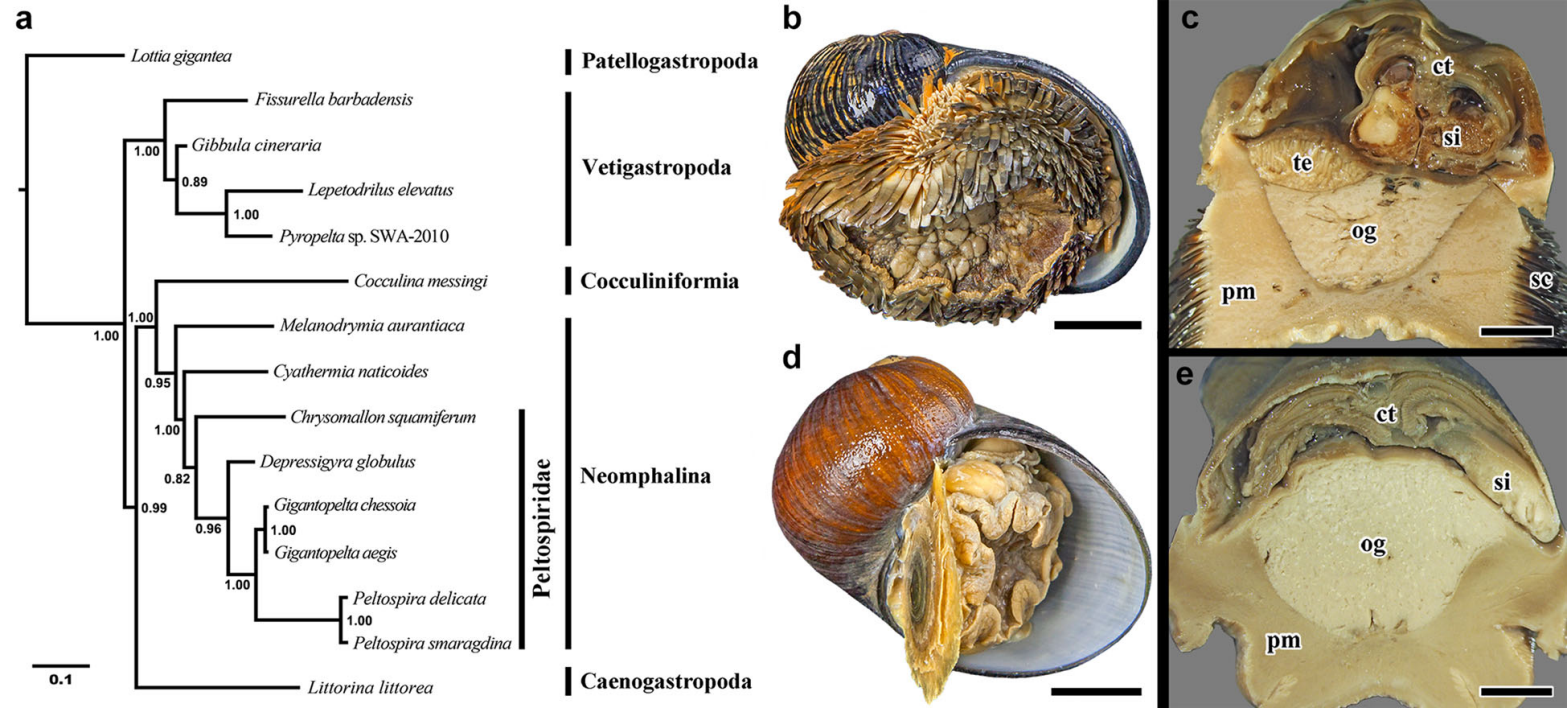
Phylogenetic context and comparison of two hydrothermal vent endemic gastropods: *Chrysomallon* (upper) and *Gigantopelta* (lower). **a.** Consensus tree reconstructed using Bayesian inference from a combined analysis of five standard markers (H3, COI, 16S, 18S, 28S), total alignment length 2753-bp. Node values indicate Bayesian posterior probability. **b.** *Chrysomallon squamiferum* (Longqi field, Southwest Indian Ridge) adult specimen in exterior ventral view and, **c.** transverse section showing internal oesophageal glad (og), scale bars 1 cm. **d.** *Gigantopelta chessoia* (E2 Segment, East Scotia Ridge, Southern Ocean) adult specimen, and **e.** transverse section showing internal oesophageal glad (og), scale bars 1 cm. Abbreviations: ct, ctenidium; pm, pedal muscle; og, oesophageal gland; sc, scales; si, blood sinus; te, testis

**Fig. 2:**
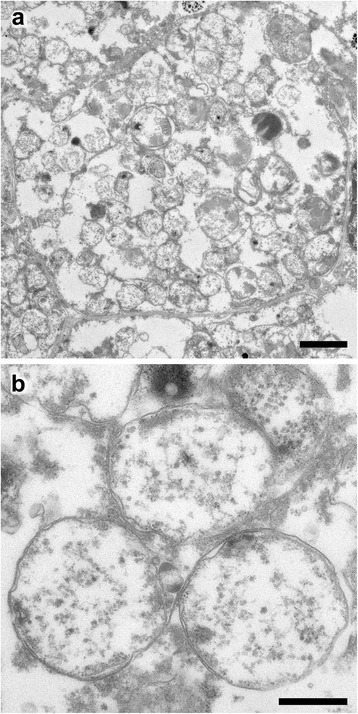
TEM micrographs of the oesophageal gland of *Gigantopelta*. **a.** An entire bacteriocyte and endosymbionts within, scale bar 2 μm; **b.** Magnification of a cluster of three endosymbionts in the oesophageal gland, scale bar 500 nm

**Fig. 3:**
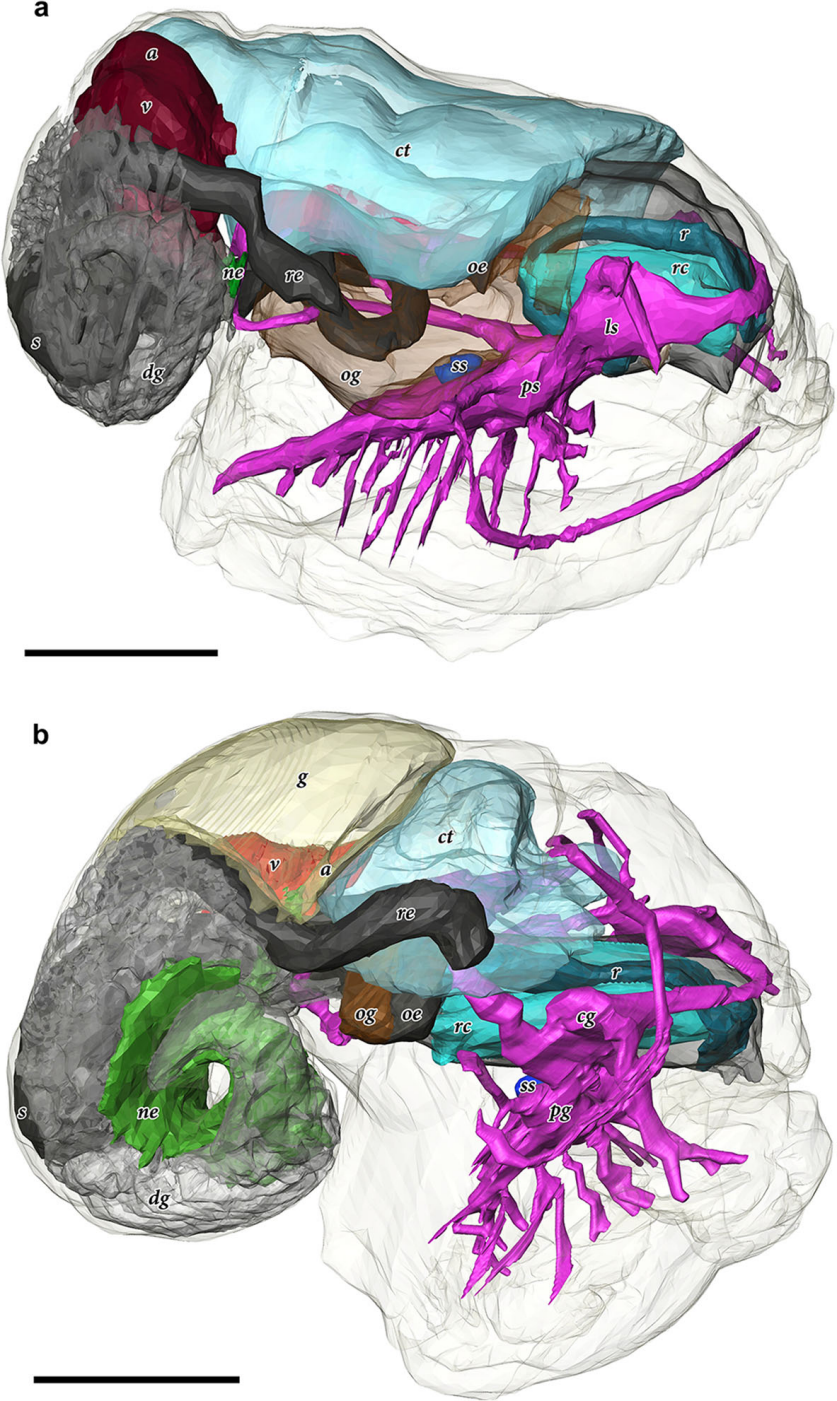
Comparative 3D tomographic reconstructions of the internal anatomy. **a.** *Chrysomallon squamiferum* (redrawn from [35]). **b.** *Gigantopelta chessoia.* Colour groups correspond to specific anatomical systems: *grey*/*black*, digestive tract; *brown*, oesophageal gland; translucent *blue*, ctenidium; *red*, heart; *yellow*, gonad; *green*, nephridium; fuchsia/*blue*, nervous and sensory systems. Abbreviations: a, auricle; cg, cerebral ganglia; ct, ctenidium; dg, digestive gland; g, gonad; ls, non-ganglionic lateral swelling [35], ne, nephridium; oe, oesophagus; og, oesophageal gland; pg, pedal ganglia; ps, non-ganglionic pedal swelling [35]; r, radula; rc, radula cartilage; re, rectum; ss, statocysts; v, ventricle

The 'scaly-foot gastropod' *Chrysomallon squamiferum*, and *Gigantopelta* spp. share key features that separate them from all other known hydrothermal vent gastropods. However, our analysis clearly demonstrated that this suite of adaptations to a vent ecosystem has been convergently acquired in the two genera (Fig. 1). Phylogenetic analysis of neomphaline gastropods demonstrated that the two genera are not sister groups. We confirmed that the oesophageal gland in *Gigantopelta chessoia* does house endosymbiotic bacteria (Fig. 2), but comparative anatomy (Fig. 3) also revealed substantial differences that belie the potential homology of these adaptive features.

In our Bayesian phylogenetic analysis, Peltospiridae was recovered as a monophyletic group (Bayesian Posterior Probability, BPP = 0.82) within the fully-supported monophyletic clade Neomphalina (BPP = 1.00) (Fig. 1). Both *Chrysomallon* and *Gigantopelta* are within the family Peltospiridae, confirming their systematic position within the family. The other peltospirid taxa, *Depressigyra* and *Peltospira* spp. retain the plesiomorphic conditions (no dermal scales, no endosymbiont-housing oesophageal gland, normal circulatory capacity), given polarity inferred from non-peltospirid neomphalines and all other gastropods [32–34]. While *Chrysomallon* is sister to all other Peltospiridae, the distinctive anatomical features of this species such as its dermal scales, unganglionated nervous system, oesophageal gland for endosymbionts, and enlarged circulatory system, are all unequivocally derived features within Neomphalina [35]. *Gigantopelta*, which appears to share some of these features, was recovered sister to *Peltospira* with full support (BPP = 1.00), and the *Gigantopelta-Peltospira* clade was sister to *Depressigyra* (BPP = 0.98). It is most parisomonious to infer that derived anatomical characters in *Gigantopelta* were acquired as independent autapomorphies, not from a common peltospirid ancestor. Despite the similarities between the two giant peltospirid genera, *Gigantopelta* and the ‘scaly-foot gastropod’ *Chrysomallon* are actually not the closest-related members among the known Peltospiridae.

**Fig. 4:**
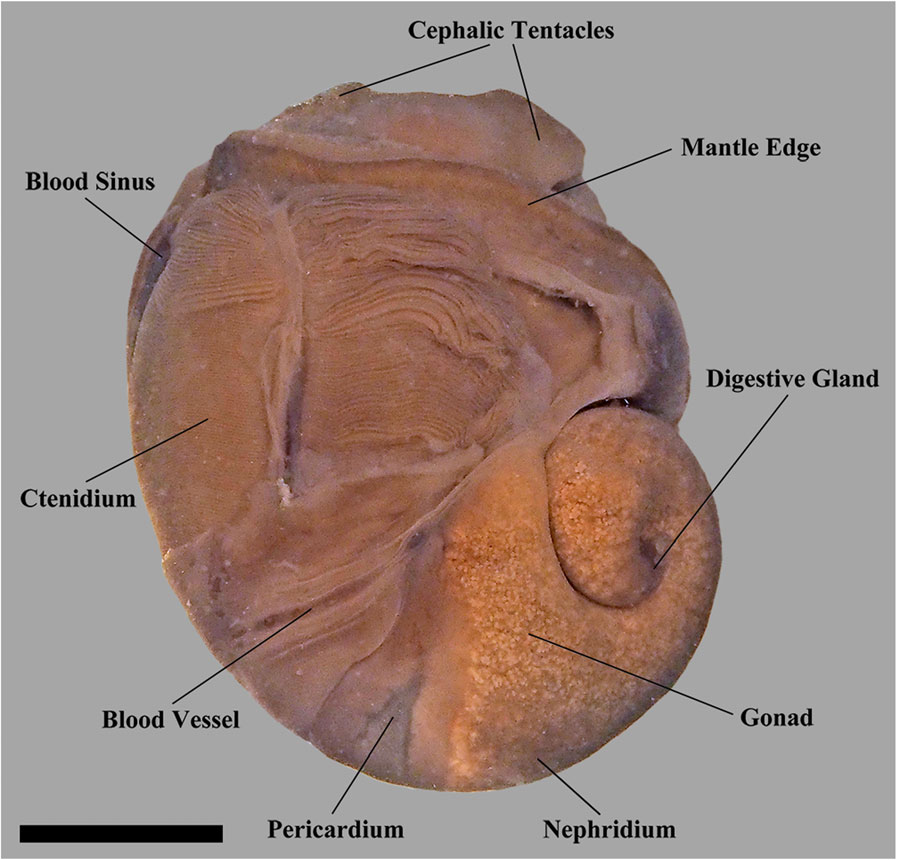
Gross anatomy of *Gigantopelta chessoia* (shell and mantle tissue partially removed). Scale bar, 1 cm

**Fig. 5:**
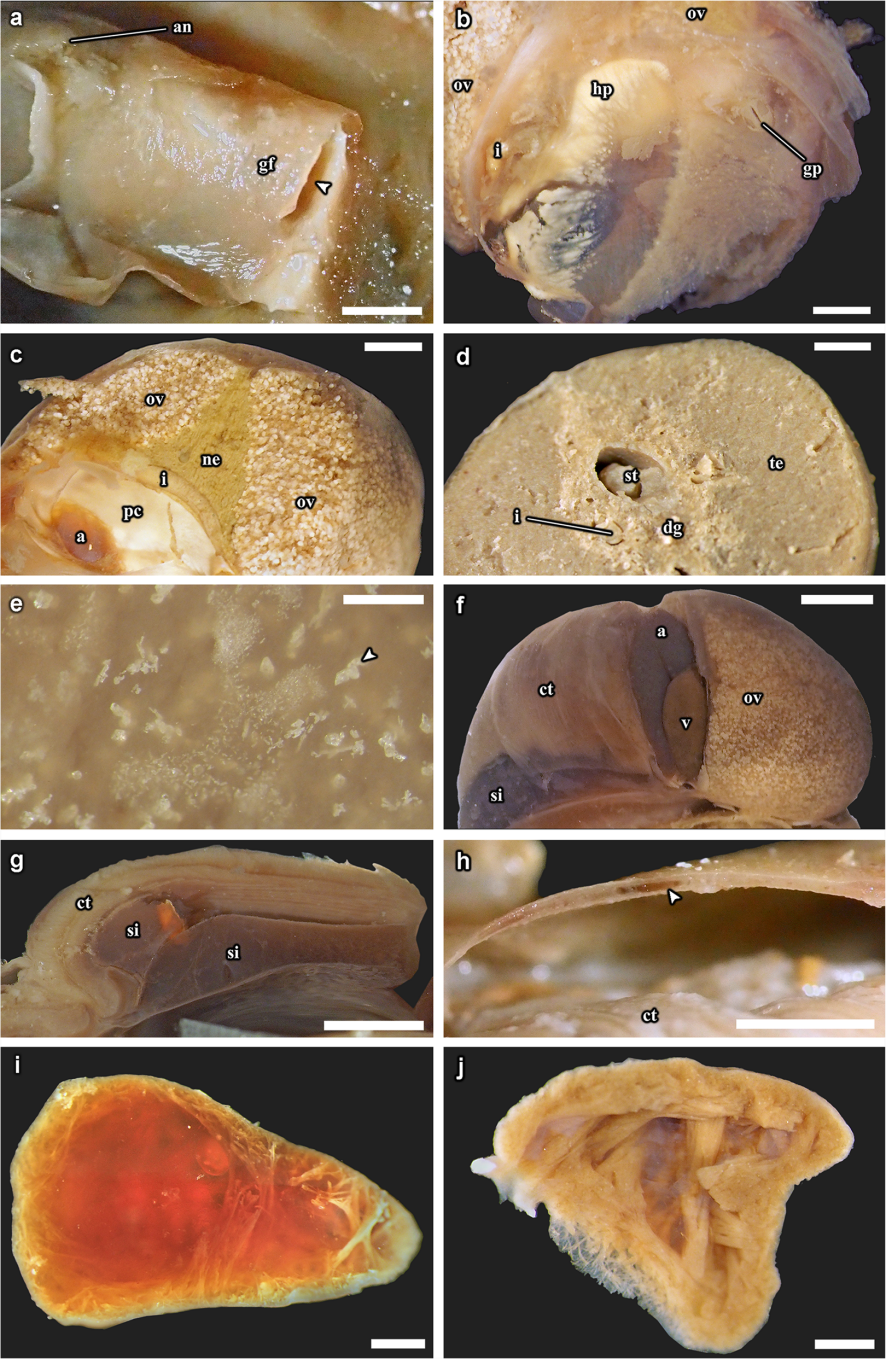
*Gigantopelta chessoia*, photographs from dissection of adult specimens. **a.** Inside face of the right-posterior part of the mantle with gonad ‘flap’ removed to reveal the gonopore. **b.** A section of the right-posterior mantle wall showing the gonad ‘flap’ and anus. Arrowhead indicates the gonad ‘flap’ opening. **c.** Transverse section through a female, showing relative positions of ovary and nephridium. **d.** Transverse section through a male, showing stomach and digestive gland. **e.** Close-up of granular crystalline material (arrowhead) within the oesophageal gland. **f.** Posterior view with the pericardium removed to reveal the auricle and the ventricle. **g.** Transverse section through the ctenidium showing the large blood sinuses underneath. **h.** Transverse section through the mantle roof showing large blood vessel (arrowhead). **i.** Sagittal section through the ventricle, with blood clot removed to show muscle bundles. **j.** Sagittal section through the auricle. Abbreviations: a, auricle; an, anus; hp, hypobranchial gland; ct, ctenidium; dg, digestive gland; gf, gonad ‘flap’; gp, gonopore; i, intestine; ne, nephridium; pe, pericardium; og, oesophageal gland; ov, ovary; s, stomach; si, blood sinus; te, testis; v, ventricle. Scale bars of **a–b, e, i–j**: 1 mm; **f–g, h**: 5 mm; **c–d**: 2 mm

**Fig. 6:**
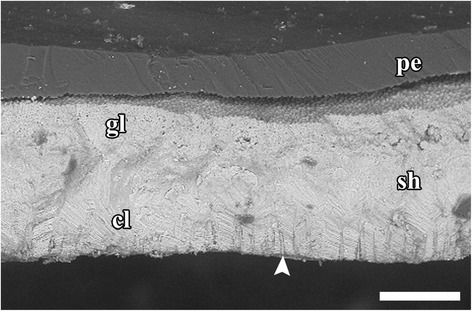
Cross section of *Gigantopelta chessoia* shell showing microstructure and proximal shell pores (*white arrowhead*) spread evenly across inner surface. Abbreviations: cl, cross lamellar layer; gl, granulose layer; pe, periostracum; sh, shell. Scale bar 100 μm

**Fig. 7:**
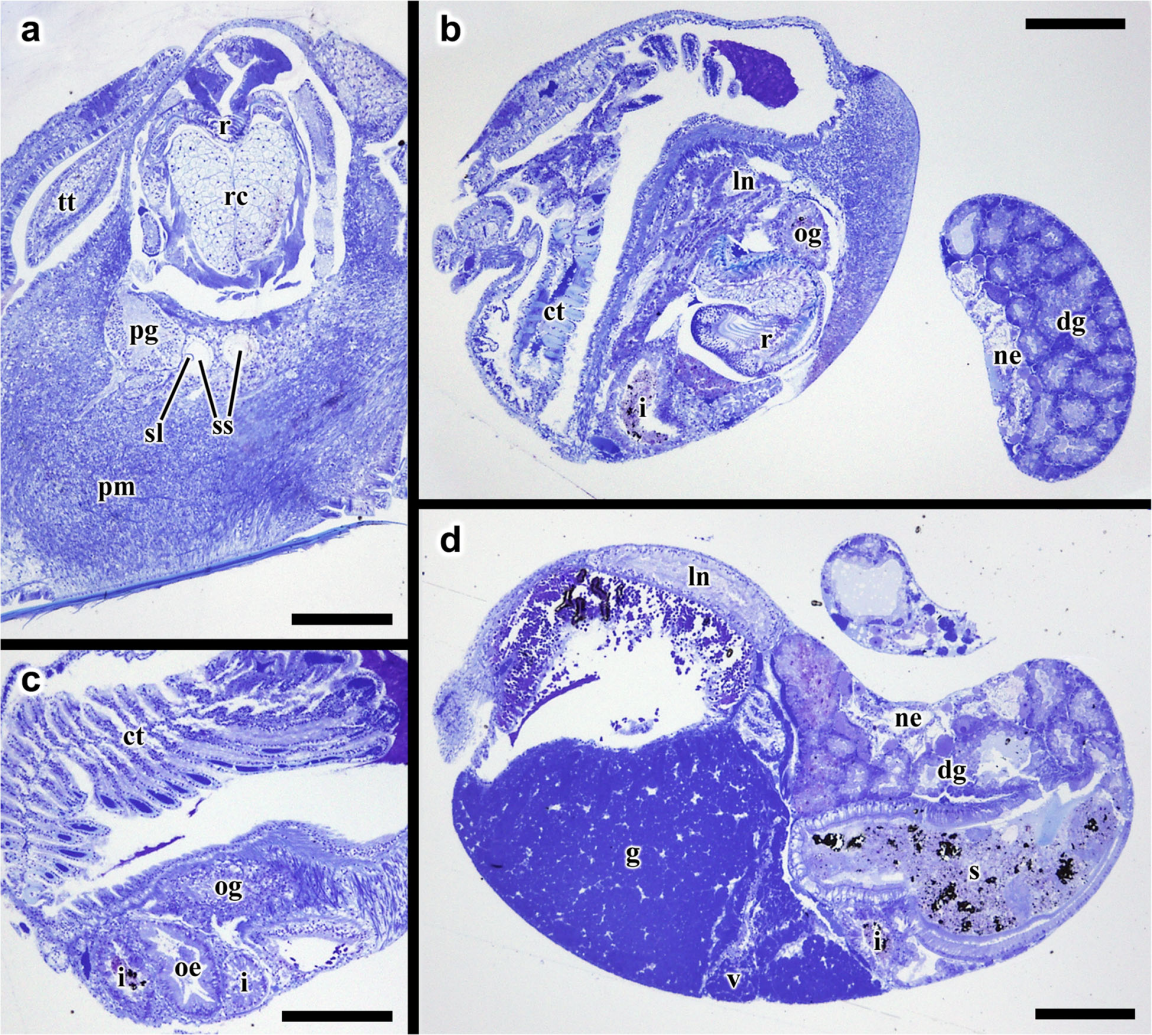
Transverse semi-thin sections from *Gigantopelta chessoia*. **a.** Posterior part of the head showing radula apparatus, pedal ganglion, and statocysts. **b.** Mid-body section showing the start of oesophageal gland. **c.** Mid-body section showing the oesophageal gland, sections through the digestive tract, and ctenidium. **d.** Section through the anterior part of the visceral sac showing the gonad, the ventricle and the digestive glands. Abbreviations: ct, ctenidium; dg, digestive gland; g, gonad; i, intestine; ln, lateral nerve cord; ne, nephridium; oe, oesophagus; og, oesophageal gland; pg, pedal ganglion; pm, pedal musculature; r, radula; rc, radula cartilage; re, rectum; s, stomach; sl, statolith; ss, statocyst; tt, cephalic tentacle; v, ventricle. Scale bars all 200 μm

**Fig. 8:**
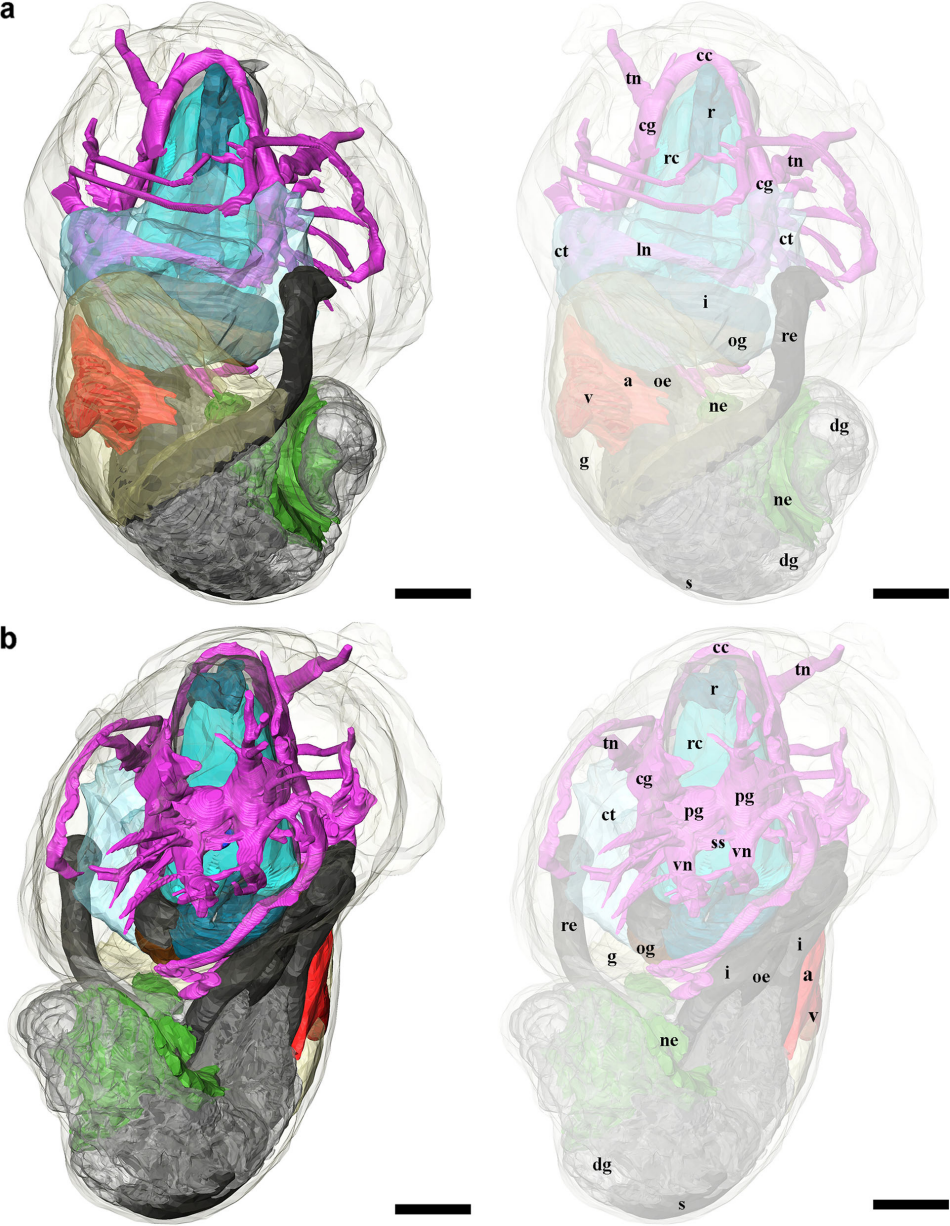
3D tomographic reconstruction of *Gigantopelta chessoia*, the full anatomical model in various views. Soft body outline (mantle and foot) shown in transparency for context. Ctenidium, anterior oesophagus, and digestive gland are rendered semi-transparent to show the structures underneath. For all parts, the tomographic model is shown to left and a second copy of the same view with labelled parts shown to right. **a.** Dorsal view; **b.** Ventral view. Colour groups correspond to specific anatomical systems: *grey*/*black*, digestive tract; *brown*, oesophageal gland; translucent *blue*, ctenidium; *red*, heart; *yellow*, gonad; *green*, nephridium; fuchsia/*blue*, nervous and sensory systems. Abbreviations: a, auricle; cc, cerebral commissure; cg, cephalic ganglion; ct, ctenidium; dg, digestive gland; g, gonad; i, intestine; ln, lateral nerve cord; ne, nephridium; oe, oesophagus; og, oesophageal gland; pg, pedal ganglion; r, radula; rc, radula cartilage; re, rectum; s, stomach; si, blood sinus; ss, statocyst; tn, tentacular nerves; v, ventricle; vn, ventral nerve cord. Scale bars, all 250 μm

**Fig. 9:**
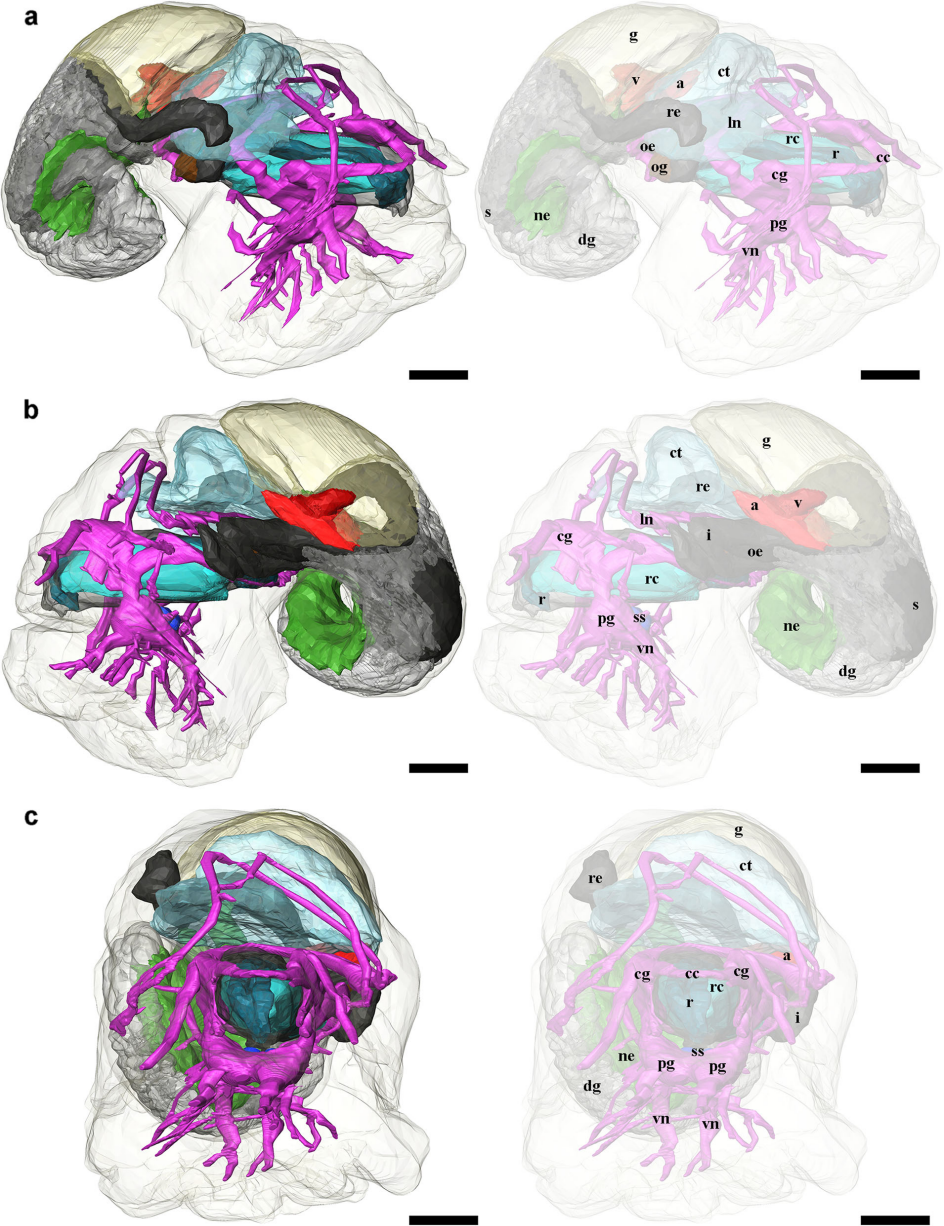
3D tomographic reconstruction of *Gigantopelta chessoia*, the full anatomical model in various views (continued). Soft body outline (mantle and foot) shown in transparency for context. Ctenidium, anterior oesophagus, and digestive gland are rendered semi-transparent to show the structures underneath. For all parts, the tomographic model is shown to left and a second copy of the same view with labelled parts shown to right. **a.** Right side view; **b.** Left side view; **c.** Anterior view. Colour groups correspond to specific anatomical systems: *grey*/*black*, digestive tract; *brown*, oesophageal gland; translucent *blue*, ctenidium; *red*, heart; *yellow*, gonad; *green*, nephridium; fuchsia/*blue*, nervous and sensory systems. Abbreviations: a, auricle; cc, cerebral commissure; cg, cephalic ganglion; ct, ctenidium; dg, digestive gland; g, gonad; i, intestine; ln, lateral nerve cord; ne, nephridium; oe, oesophagus; og, oesophageal gland; pg, pedal ganglion; r, radula; rc, radula cartilage; re, rectum; s, stomach; si, blood sinus; ss, statocyst; tn, tentacular nerves; v, ventricle; vn, ventral nerve cord. Scale bars, all 250 μm

**Fig. 10:**
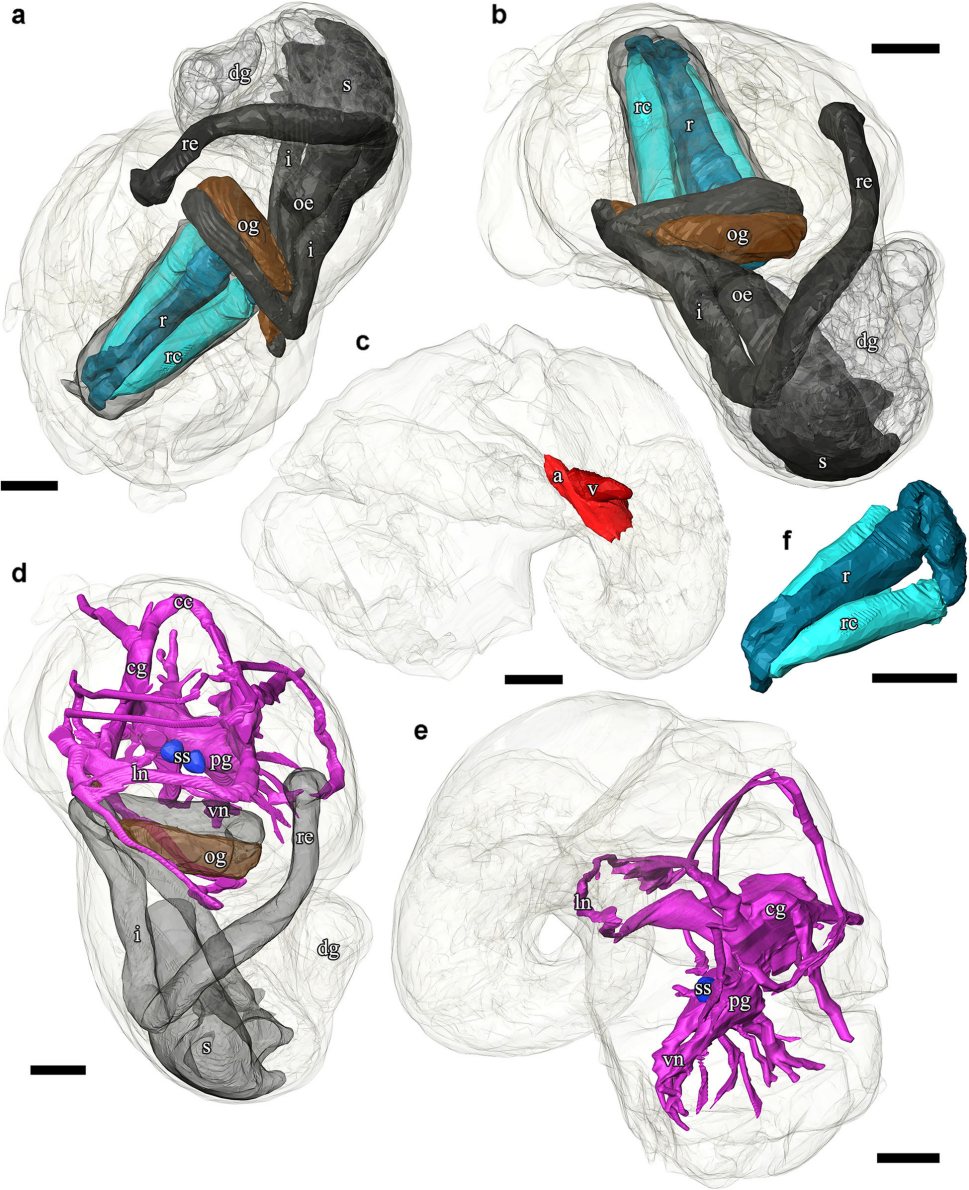
3D tomographic reconstruction of *Gigantopelta chessoia*, organ groups. Soft body outline (mantle and foot) shown in transparency. Anterior oesophagus, and digestive gland are rendered semi-transparent to show the structures underneath. **a-b.** Digestive system; **c.** Heart; **d.** Nervous system with mid- and hindgut shown semi-transparent for positional reference; **e.** Side view of the nervous system; **f.** Radula and radula cartilage. Abbreviations: a, auricle; cc, cerebral commissure; cg, cephalic ganglion; dg, digestive gland; i, intestine; ln, lateral nerve cord; ne, nephridium; oe, oesophagus; og, oesophageal gland; pg, pedal ganglion; r, radula; rc, radula cartilage; re, rectum; s, stomach; ss, statocyst; v, ventricle; vn, ventral nerve cord. Scale bars, all 250 μm

Features of the shell, radula, operculum and general configuration of the soft parts of *Gigantopelta chessoia* and the congener *G. aegis* were described by Chen et al. [16]. The following description is drawn from observations of *G. chessoia* using dissection of adult specimens (Fig. 4), including soft parts (Fig. 5) and shell (Fig. 6), as well as and histology of a juvenile specimen (Fig. 7), with tomographic reconstructions of the juvenile organ systems (Figs. 8, 9 and 10). The ‘juvenile’ specimen examined in detail via serial sectioning had an intact gonad, however the apparently ontogenetic allometry of the oesophageal gland led us to maintain an explicit differentiation of some ‘juvenile’ characters, meaning they were observed in the histology of tomography of the figured specimen. The features described here are generally applicable across ontogeny unless specifically stated to apply differentially to juveniles and adults. The authors have also examined adult specimens of the congener *G. aegis*, which is similar in terms of gross anatomy (CC, JDS, unpub. obs.), and some comparative remarks are included where the two species differ. Anatomical descriptions of specimens of both species are limited to observations from material fixed in formalin (*G. chessoia n* = 5, *G. aegis n* = 7, both fixed in 10% formalin in phosphate-buffered seawater; [16]). The full interactive 3D anatomical model is provided as a PDF file in Additional file 1.

### External morphology and general features

*Gigantopelta chessoia* is a loosely coiled gastropod, the head-foot and visceral mass occupy approximately two whorls (Fig. 4). The snout is thick and broad, expanding slightly towards the distal end. A pair of long and prominent cephalic tentacles is present, about twice the length of the snout, broad at the base and slowly tapering to a fine tip. No eyes are present (confirmed via histology) and the cephalic tentacles are of equal size in both sexes. There are no copulatory organs on the head-foot. The head is clearly separated from the rest of the head-foot by a horizontal flap formed by the epipodium. A large multispiral operculum is present at the metapodium in both adults and juveniles, and fits well into the aperture when the animal is retracted into the shell. The foot is has no tentacles or other appendages on the exposed surface, but a single row of epipodial tentacles are present under the operculum, surrounding the opercular attachment. The head-foot flesh is generally pinkish in colouration, with patches of purplish blue when alive. The shell muscle is U-shaped, with two thick lateral parts on left and right, connected posteriorly by a narrow attachment. The mantle edge is thick and smooth, the mantle cavity is moderate in size, reaching two-thirds of the body length. The mantle wall is often embedded with unidentified crystalline deposits in *G. aegis*, although this was not seen in specimens of *G. chessoia* preserved with the same methods. The ctenidium is rather large and occupies the left side of the mantle cavity. In adults, a greatly enlarged oesophageal gland occupies the floor of mantle cavity as a smooth hump. The main visceral mass occupies approximately 1.5 whorls (Figs. 4 and 9).

The hypobranchial gland is visible in the dorsal right aspect of the mantle tissue, as an elongate pale diffuse glandular material (the gland is visible in the proximal mantle tissue in Fig. 5b; it is not visible in Fig. 4 because the mantle tissue was removed to reveal the ctenidium).

### Shell microsculpture

The thin outer layer of the shell had a finely granular microstructure, while the thicker inner layer is composed of a cross-lamellar structure as is typical of Peltospiridae [36, 37]. Numerous narrow shell pores are present at the inner surface of the shell, these open to the inside and gradually taper towards the outer surface though they do not appear to infiltrate the outer granular layer (Fig. 6). Similar proximal shell pores are apparently commonplace but not universal within the clade Neomphalina [34, 38]. The shell microstructure and the presence of inward facing shell pores are similar in *Gigantopelta chessoia* and *G. aegis* (CC unpub. obs.). The periostracum of *G. aegis* is overlaid with a thick layer of rusty deposits, while *G. chessoia* lacked this (as reported in [16]).

### Digestive and excretory systems

A ventral mouth opening on the snout leads to buccal cavity containing the rhipidoglossate radula. The inner lip is composed of columnar epithelial cells covered with a thin cuticle. We could not locate any discrete jaws or salivary glands. The radula has a width:length ratio of about 1:10 in the specimen used for tomographic reconstruction. The radula is supported by a single pair of prominent cartilages, in contact with each other anteriorly and separating posteriorly. The radula sac is posterior to the cartilages and ventral to the buccal mass (Figs. 7 and 10: radula, r; radula cartilage, rc). The radula ribbon emerges folded and is straightened at the cartilages. A portion of the oesophagus enlarges to a blind-ended oesophageal gland, extremely hypertrophied in the adults (Fig. 1) but not so in the juveniles (Figs. 7 and 10: oesophageal gland, og). The oesophageal gland in adults has a generally uniform texture except a complex network of semi-enclosed tubes embedded within the gland mass, as well as numerous crystals of unknown nature (Fig. 5e).

The digestive tract is relatively small and forms a simple loop (Fig. 10). The oesophagus runs posteriorly straight into a distinct stomach in the midgut without looping, running underneath the pericardium and the gonad on the way. The stomach is clearly distinct from the rest of the digestive tract, being much wider than the foregut or hindgut. No gastric shields or styles were present. At least three ducts connect the stomach to the digestive gland; the digestive gland extends posteriorly to fill most of the apex (Figs. 5 and 8: digestive gland, dg). While apical in position, distal to the stomach and the posterior-most part of the digestive loop, the digestive gland can appear overgrown by gonad in adults (Fig. 6d). The posterior intestine emerges from the right-posterior end of the stomach and proceeds anteriorly into the oesophageal gland. In adults the posterior intestine is very fine (maximally 1.2 mm in width for a specimen 36 mm shell length; Fig. 5c) and its integument thin and fragile, so where the digestive tract is embedded in the hypertrophied oesophageal gland the path of the hindgut was impossible to accurately trace via dissection. In juveniles, where the oesophageal gland is proportionately much smaller, the hindgut intestine exits the stomach running anteriorly toward the mouth, then loops back to the posterior just before reaching the buccal mass to return posteriorly towards the stomach, staying entirely ventral of the pericardium and the gonads. Slightly anterior to the stomach, the digestive loop turns anteriorly again for a final stretch running along the mantle wall, entering into the mantle and emerging as the rectum. Thus the rectum does not pass through the pericardium. The anal opening is located on the right side of the body on the mantle wall, dorsal of the genital opening. The digestive tract posterior of the stomach is often filled by a chalky material. This was seen in specimens across all sizes and may represent sulphur granules produced by the endosymbiont and represent a way for detoxing hydrogen sulphide.

The nephridium is a mass of glandular tissue that starts posterior to the oesophageal gland, and extends into the visceral sac. It is of considerable size and occupies the ventral side of the visceral sac, ending just before reaching the apex (Figs. 7, 8 and 9; nephridium, ne). In adults, the gonad surrounds the nephridium on both sides (Fig. 9a).

### Circulatory system

A single, moderately large left ctenidium (Figs. 4, 7c and 8) terminates at the posterior end of the mantle cavity. The posterior end is fused to the mantle roof. The ctenidium occupied 7.9% of the body volume in the serially sectioned specimen. The gill is bipectinate, densely packed with fine gill filaments on both sides. The filaments are supported by skeletal rods. Sensory bursicles could not be confirmed. Two to three prominent and semi-enclosed blood sinuses are present under the left side of the gill. The blood sinuses in *Gigantopelta* appear to be static and only present under the gill. There is a complex network of large enclosed blood vessels that run through the mantle tissue (Fig. 5h).

The heart of *Gigantopelta* is monotocardian, composed of a well separated ventricle and auricle, both of which have closed, muscular walls (Fig. 5f, i, j: auricle, a, ventricle v). The auricle is large and of a curved triangular shape (Fig. 5i), positioned to the posterior of the ctenidium, and the ventricle is of slightly smaller size (Fig. 5j) left and ventral of the auricle contacting the auricle’s convex side (Fig. 5f). A large pericardium encloses the heart with tissue fused to the mantle roof. The auricle has walls and interior space made of crossing muscle fibres, and a large muscle bundle divides it into two unequal portions (Fig. 5i). The ventricle has more muscular walls and even thicker muscle bundles than the auricle (Fig. 5j), and occupied approximately 1.9% of the body volume in the serially sectioned specimen (Fig. 10c). In dissected specimens, the auricle is larger than the ventricle; in the serially-sectioned juvenile specimen the auricle was clearly damaged so more detailed quantitative estimates about comparative volume are not available. The haemocoel is jelly-like and of a pale blue colouration in fixed specimens, but the muscles of the heart, particularly the auricle are dark red. The heart in *G. aegis* is much paler in colouration and appear to have generally thinner walls. The auricle, especially, is smaller in size and much softer in texture (with less muscular walls) than *G. chessoia*.

### Reproductive system

*Gigantopelta chessoia* is gonochorostic, the level of development of either testes or ovary varied among individuals of each sex. (All known specimens of both *G. chessoia* and *G. aegis* were collected in several events in different years but all in austral summer, November-December.) The testes were only observed in specimens that were preserved in ethanol but appear relatively paler than the ovae; the ovary is pinkish-brown. The gonad is largely posterior of the pericardium, except a thin leaf of mature gonad extending to dorsally overlap the pericardium (Fig. 9c). In *G. aegis*, this extension was not present and the gonad was entirely contained posterior of the pericardium (Chen et al. 2015a: Fig. 7). The gonad is connected to a pouch within the mantle roof (Fig. 5a) via a gonopore presenting as a simple slit on the proximal right mantle wall (Fig. 5b). A pocket surrounds the gonopore and extends along the right mantle wall. Within the mantle-cavity, a cresent-shaped flap opening into this pouch is located anterior to the anus (Fig. 8a). The pouch is located on the left side body; it takes up considerable space and is visible as thicker tissue sometimes containing gonad material embedded within the left side and posterior mantle tissue, tilting toward the posterior where it connects to the visceral mass via the gonopore. The pouch within *G. aegis* is lined with muscular folds that make the pouch or flap appear extensible, though the direction of expansion is unclear. The gonoduct is not associated with any specialised copulatory organs or appendages, the gonad is well contained within the mantle and there is no penis modified from cephalic tentacles. No external sexual dimorphism could therefore be detected. Each of the individuals examined had only a single type of gonad present. The serially sectioned juvenile specimen already had a sizeable gonad (Figs. 7d, 8 and 9), taking up 11.4% of the body volume in the same position as the adults.

### Nervous system and sensory structures

Our descriptions follow the standard ontology proposed by Richter et al. [39]. The nervous system is rather voluminous (Figs. 8, 9 and 10) and occupies approximately 3.8% of body volume in the serially sectioned juvenile specimen, proportionately smaller in adults. Both cephalic tentacles are innervated with prominent tentacular nerves. The nervous system is ganglionic, with an oesophageal nerve ring containing distinct cerebral and pedal ganglia pairs.

The pair of cerebral ganglia are large, flat and round, the edge of the discs oriented anterior-posteriorly. These are interconnected by a horseshoe-shaped cerebral commissure. The medullary cords (sensu [39]) in the cephalic tentacles emerge from the central anterior margin of the cerebral ganglia. Other neural projections emerge from the anterio-ventral side that innervate the buccal mass though no ganglia could be identified. Directly below the cerebral ganglia, slightly posterior to the buccal nerve projections, are the pedal ganglia. The two pedal and cerebral ganglion pairs are not fused but closely connected. The pedal ganglia are teardrop-shaped, tapering to the posterior and connected to the medullary ventral (pedal) nerve cords which extend ventrally and laterally into the foot with numerous large branches. There are at least two lateral interconnectives joining the two ventral nerve cords within the foot muscle, but the posterior ends of the ventral nerve cords are not joined. The pedal nerve cords are fully embedded within the foot muscle; these nerves are very large and visible without magnification in adult specimens.

On the distal side of each pedal ganglion, a distinct interconnective extends dorsally to connect with the lateral nerve cord immediately posterior to the cerebral ganglion. This point on the lateral nerve cord is positionally homologous to the pleural ganglia in other molluscs but there is no discrete ganglion in this position in *G. chessoia*. At the same point on the lateral nerve cords, another nerve emerges extending dorsally into the mantle edge.

The nervous system is torted, resulting in a typical figure-of-eight shape in the lateral nerve cords. The body left side lateral nerve emerged from the posterior aspect of the cerebral ganglion; immediately behind the ganglion is a swelling equivalent to the pleural position, with distal lateral connectives to the pedal ganglion and mantel nerves as described. Posterior to the pleural area, the nerve cord extends posteriorly and tilting to the right, passing ventrally under the intestine and oesophageal gland. Posterior to the oesophageal gland the lateral nerve loop turns to the dorsal and comes forward over the intestine and oesophageal gland, passing under the gill. There is a large neural mass at the base of the gill rachis which is positionally equivalent to the position of the osphradial ganglion in other taxa though no pigmentation corresponding to an ‘osphradium’ was consistently observed on the gill epithelium in preserved specimens. Another nerve emerges anterior from this neural swelling and extends to the right as another mantle nerve. The lateral nerve cord loop continues after the point of this neural swelling (positionally similar to the osphradium in other taxa), turning to the right and crossing the body to then join the right side cerebral ganglion in a pleural-area swelling nearly symmetrical to the left side.

A pair of statocysts are located medially, more or less central in the body (Fig. 10d: statocysts, ss). These are situated on the inner dorsal side of pedal ganglia and apparently innervated by them. Each statocyst contains a single statolith (Fig. 6a).

## Discussion

The phylogenetic context suggests independent evolutionary origins of the respective autapomorphies of the two genera *Chrysomallon* and *Gigantopelta*; a comparative anatomical approach can illuminate which aspects have a common origin and the potential evolutionary constraint of adaptations to extreme conditions at hydrothermal vents. The anatomy of *Gigantopelta* broadly agrees with other members of Neomphalina, having a single left bipectinate ctenidium, non-papillate tentacles, a single left auricle, rectum that does not penetrate the pericardium, statocysts with statolith, and one pair of radula cartilages [32, 34], but with a number of distinctive modifications (Fig. 3). Here, we consider five key character sets in *Gigantopelta* that were previously identified as adaptive features in *Chrysomallon* [35]: armature, nervous system, endosymbiont-housing oesophageal gland, circulatory system, and reproduction (Table 1).

**Table 1:** Characteristics of the giant peltospirids, *Gigantopelta* and *Chrysomallon*, in comparison to other species that retain plesiomorphic character states for the family Peltospiridae (*Peltospira delicata*) and the parent clade Neomphalina (*Melanodrymia aurantica*)

	Neomphalina					
		Peltospiridae				
	*Melanodrymia aurantica*	*Peltospira delicata*	*Gigantopelta chessoia*		*Chrysomallon squamiferum*	
Body size	adult 2 mm	adult 6 mm	juvenile (2 mm)	adult (50 mm)	juvenile (2.2 mm)	adult (45 mm)
Dermal armature	none	none	none		scales in few rows	dense and asymmetric scales
Nervous system	ganglionate	ganglionate	ganglionate		non-ganglionate	
Oesophageal gland	symmetrical foregut pouches without glandular material	small, symmetrical foregut glands	fused, enlarged gland, occupying 0.6% of visceral mass volume	fused, enlarged gland, increasing allometrically up to 9% of visceral mass volume	fused, enlarged gland, occupying 9% of visceral mass volume	fused, enlarged gland, increasing isometrically with growth
Heart	heart not enlarged (ventricle 0.10 mm in animal length 2.1 mm)	heart not enlarged (ventricle 0.65 mm in animal length 6 mm)	heart greatly enlarged (ventricle 0.42 mm in animal length 2.0 mm)	heart greatly enlarged and muscular (ventricle 6 mm)	heart greatly enlarged and muscular (ventricle 0.64 mm in animal length 2.2 mm)	heart greatly enlarged and muscular (ventricle 8 mm)
Blood sinuses	thin	thin	large, but few and fixed position		many large and mobile blood sinuses	
Reproduction	gonochoristic	gonochoristic	gonochoristic, fully developed gonad at body size 2.0 mm	gonochoristic	no gonad present at body size 2.2 mm	simultaneous hermaphrodite

These characters are drawn from published observations and new data herein [32, 35, 49]. Separate observations of juvenile and adult specimens for the giant peltospirids provides insights into heterochronic shifts, particularly the late development of the endosymbiont-housing oesophageal gland in *Gigantopelta*

The most striking features of the ‘scaly-foot gastropod’ *Chrysomallon squamiferum* is the dermal scales on its foot, grown as mineralised outpockets of epithelium that may originate from structures similar to the dermal tentacles seen in all other known Peltospiridae [20]. At the Southwest Indian Ridge, *Chrysomallon* and *Gigantopelta* co-occur, with colonies of snails side-by-side [16]. While the shell of *Gigantopelta* is robust and well calcified compared to other large vent endemic gastropods such as *Alviniconcha* [40, 41], the foot epithelium of *Gigantopelta* is normal and unarmoured.

The nervous systems of these two genera show some critically important differences. Descriptions of invertebrate nervous systems are hampered by terminology that sometimes does not facilitate comparisons among groups [42]. In *Chrysomallon*, the nervous system is unganglionated (specifically meaning it lacked any areas were the neuropil of the medullary cords was clearly surrounded in three dimensions by a cell cortex of neural somata [39]), reduced into a series of nonetheless massive medullary cords [35]. This lack of neural organisation could be interpreted as indicative of a lack of any higher processing in the snail, or that complex behaviour does not provide any selective advantage to an animal primarily dependent on maintaining an optimal environment for its endosymbiont microbes. The ventral nerve cords of *Chrysomallon* are so enlarged, they sit outside the foot musculature, within the visceral mass, in direct contact with the oesophageal gland [35]. By contrast, the nervous system of *Gigantopelta* possesses the true ganglia of a typical molluscan nervous system and a neural architecture similar to that described in some vetigastropods [43].

One striking feature shared by *Gigantopelta* and *Chrysomallon* is their large size. Yet most vent-endemic gastropods that have acquired endosymbionts have a tendency towards gigantism, including *Alviniconcha* and *Ifremeria* in Provannidae [21].

All other known hydrothermal vent-endemic molluscs that house chemosymbionts, do so in their gills [28]. Although the oesophageal gland is a common structure among the plesiomorphic gastropod clades such as Vetigastropoda, Neomphalina, and Cocculiniformia [44], only in *Gigantopelta* and *Chrysomallon* is the oesophageal gland known to be hypertrophied to fill the entire ventral side of mantle cavity [18, 35]. Though we cannot entirely dismiss that other peltospirids may host a low density of bacteria perhaps for detoxification, it is clear that their glands are not enlarged and do not play important nutritional role [32]. It is worth mentioning that large monoplacophoran species also have hypertrophied oesophageal glands, although all extant monoplacophorans studies so far have proved to be detritivorous [45].

The digestive tract of *Gigantopelta* is similar in length to *Chrysomallon*, being short and consisting of a single loop, consistent with reliance on bacteria for primary nutrition. In *G. chessoia*, the oesophageal gland is proportionately much smaller in juveniles (only 0.6% body volume) compared to adults, indicating a shift of diet during ontogeny. This heterochrony is in stark contrast to *Chrysomallon*, in which the oesophageal gland scales isometrically with growth (9% body volume [35]). The radula (1.4% body volume) and radula cartilages (2.6% body volume) are both much larger in *Gigantopelta* compared to *Chrysomallon* (0.4% and 0.8% body volume, respectively) at juvenile stages. While *Chrysomallon* is an obligate symbiotroph throughout post-settlement life, *Gigantopelta* may be a mixotroph in juvenile life and shifting to obligate symbiotrophy as an adult.

Observations via TEM confirmed that *Gigantopelta chessoia* houses intracellular bacteria in the enlarged oesophageal gland (microbes approximately 1 μm cell size, Fig. 2). The most common type is likely sulfur-oxidising, as we could not observe any membrane stacks that would be characteristic of methane-oxidising bacteria [46, 47]. Characterisations using 16S rRNA have shown that the most common endosymbiont in this tissue is a ɣ-proteobacteria (Jane Heywood, pers. comm.). In TEM we also observed possible additional methane-oxidising bacteria (approximately 1.5 μm cell size) occurring at low density. *Chrysomallon* has an oesophageal gland housing sulfur-oxidising ɣ-proteobacteria (cell size approximately 1.5 μm) living at high density [24]. The presence of bacteriocytes within the oesophageal gland of *Gigantopelta*, in context of a hydrothermal vent endemic lifestyle, is strong evidence that this part of the body is specialised to promote chemoautotrophic endosymbionts. The oesophageal gland tissue is much denser in *Chrysomallon*, and the connection of the oesophageal gland to the circulatory system and nervous system is different between the two genera (Fig. 1).

All other vent molluscs with endosymbionts house them on the gill surface, in constant contact with vent fluid. An oesophageal gland is a closed sac and thus the host must supply resources through its bloodstream, much like the trophosome in siboglinid tubeworms [28, 31, 48]. The whole circulatory system in both *Gigantopelta* and *Chrysomallon* is enlarged, with a disproportionately large and muscular heart, closed blood vessels and massive blood sinuses. The hearts in small individuals of these two genera are three to six times larger than in other neomphaline gastropods of similar size [32, 49] (Table 1), as well as remarkably muscular compared to all other gastropods, formed of massive intercrossing muscle bundles capable of active pumping [35]. Yet there are important differences between the two lineages. The gill in *Gigantopelta* is proportionately quite large, but relatively smaller in size compared to *Chrysomallon* (15.5% of body volume, compared to 7.9% in *Gigantopelta*), which also has a more enlarged mantle space ([35]: Fig. 1). Although the circulatory system in *Chrysomallon* is mostly closed, the prominent blood sinuses appear to be transient, and occur in different areas of the body in different individuals. By contrast, the blood sinuses of *Gigantopelta* appear to be static, and consistently occur under the left side of the ctenidium (Fig. 1). In both genera, this large blood volume is interpreted as adaptive to supply the endosymbionts with nutrients required for chemosynthesis, such as hydrogen sulfide and oxygen, but this is relatively more developed in *Chrysomallon* than *Gigantopelta.*

*Chrysomallon* is a simultaneous hermaphrodite [35], but all other members of Peltospiridae including *Gigantopelta* are gonochoristic [32, 34]. In *Gigantopelta* the gonad is mostly on the left side of the body, though connecting into a distinctive pouch embedded within the right body mantle, whereas in *Chrysomallon* the gonad mass is to the right side and the male reproductive system includes complex twisted ducts. Neither *Gigantopelta* nor *Chrysomallon* have specialised cephalic copulatory appendages, although the expandable pocket outside the gonopore seen in *G. aegis* may represent a copulatory organ. Sexual maturity is present at smaller body sizes in *Gigantopelta*, but not *Chrysomallon*, indicating different strategies to optimise fecundity.

## Conclusions

Anatomy is a key aspect of understanding organism function, and provides insights to adaptive pathways that cannot be inferred from molecular phylogenetic approaches alone. There are distinctive features of these two hydrothermal vent endemic genera that appear genrally similar, but detailed consideration of comparative anatomy shows substantial differences in every important aspect. The enlarged oesophageal gland in these gastropods is a key adaptation to optimising microbial chemosymbiosis, and other major organ systems also show modifications to support this co-option. Yet the nature of these adaptations and the configuration of organ systems do differ substantially in the two genera.

Autapomorphies and heterochronic shifts that differentiate the two lineages, are evidence of independent and convergent pathways to acquire a similar mode of life. As the two genera are not sister-taxa, a single common origin for these features would require the less plausible scenario that an enlarged oesophageal gland and giant size are plesiomorphic among peltospirids, and the other genera have all lost these characters. Within the short history of this family, adaptive novelties originated independently in two lineages for housing internal endosymbionts and for gigantism.

Recent hydrothermal-vent taxa mainly date to Cenozoic radiations [11]. The common ancestor of Peltospiridae was probably Late Cretaceous in age, with Neomphalina probably dating to Middle Jurassic, from both molecular clock reconstruction [50] and the fossil record [51]. *Chrysomallon* and *Gigantopelta* independently acquired convergent features specialised for a vent habitat, such as an enlarged oesophageal gland to house endosymbionts and hypertrophied circulatory system to supply nutrients to them, which are absent in other neomphalines (Table 1). However, as *Gigantopelta* is less dramatically derived than *Chrysomallon* and only possesses a subset of its modifications, we infer its adaptive trajectory may have begun more recently. Housing endosymbionts promotes gigantism [21, 52].

Housing them internally appears to promote additional adaptations in gastropods such as fusion of the nervous system and increased circulatory capacity. What appear to be the holobiont’s adaptations to an extreme environment, may also be interpreted as driven by optimising bacteria’s access to vent nutrients. By comparing *Gigantopelta* and *Chrysomallon*, we show that metazoans are capable of repeatedly evolving close-knit relationships with chemoautotrophic bacteria, achieving the same end-product through parallel evolution.

## Methods

### Specimen material

We examined material of both species in the genus *Gigantopelta*: *G. chessoia* occurs on the East Scotia Ridge (56° 05.31′ S, 30° 19.10′ W, depth 2644 m; collected on RRS *James Cook* expedition JC80), *G. aegis* at Longqi vent field (37° 47.03′ S 49° 38.97′ E, depth 2785 m; RRS *James Cook* expedition JC67). There are only three reported collections of these two species. Anatomical descriptions of *G. chessoia* herein are possible through material re-collected from the type locality ([16]), now deposited at the California Academy of Sciences (CASIZ 220235).

Each of the two species has been reported only from its type locality, and collections have been reported from one expedition from *G. aegis* and two occasions for *G. chessoia*; while high local abundance enabled the collection of relatively large numbers of specimens, it is relevant to summarise the limitations of presently available material.

*Gigantopelta chessoia* was initially discovered on-board RRS *James Cook* cruise JC42 in 2010, on segments E2 (type locality) and E9 of the East Scotia Ridge (Chen et al., 2015a). Approximately 1400 specimens were collected, of which about 40% were fixed and stored in 99% ethanol, 30% were fixed in 10% buffered formalin, and 20% were frozen in−80 °C. Segment E2 was revisited in 2012 during the RRS *James Cook* cruise JC80. During that cruise approximately 500 specimens were collected, of which about 40% were preserved in 99% ethanol, 30% of material in 10% buffered formalin, and 20% in−80 °C freezer. A subset of the−80 °C frozen specimens from both cruises were subjected to stable isotope analyses [26].

*Gigantopelta aegis* has only been collected once, during the RRS *James Cook* cruise JC67 from the type locality, Longqi vent field in the Southwest Indian Ridge [16]. Approximately 900 specimens were collected, of which about 45% were fixed and stored in 99% ethanol, 40% in 10% buffered formalin, and 15% in−80 °C freezer.

The Longqi vent field is currently under a license for mining exploration granted by the International Seabed Authority to the China Ocean Mineral Resources Research and Development Association. Additional collections have been made and reported in the popular press that remain unpublished in the scientific literature.

The type series of each of the two species is housed in public repositories while the remainder at time of writing are still held by the lab groups associated with the cruises, including mainly National Oceanography Centre Southampton, British Antarctic Survey, University of Oxford, and University of Newcastle. The type series of each species includes a holotype (both in 99% ethanol, NHMUK) and paratype specimens distributed among 4 public museums [16]. Additional specimens of *G. chessoia* re-collected from the type locality during cruise JC80, and used for anatomical descriptions here, have been deposited in the California Academy of Sciences (CASIZ) and the Zoologische Staatssammlung Munchen (ZSM).

### Molecular phylogeny

In order to test the hypothesis that the two genera, *Gigantopelta* spp. and the scaly-foot gastropod *Chrysomallon squamiferum*, are sister-groups, we assembled a molecular dataset for these three species and other selected relevant candidate sister-taxa, chiefly *Depressigyra* and *Peltospira*. Our analysis included eight ingroup taxa in Neomphalina (*Chrysomallon squamiferum*, *Cyathermia naticoides*, *Depressigyra globulus*, *Gigantopelta chessoia*, *G. aegis*, *Melanodrymia aurantiaca*, *Peltospira delicata*, *P. smaragdina*), and seven additional species spanning gastropod diversity, rooted with a patellogastropod as the outgroup (Patellogastropoda: *Lottia gigantea*; Vetigastropoda: *Fissurella barbadensis*, *Gibbula cineraria*, *Lepetodrilus elevatus*, *Pyropelta* sp.; Cocculiniformia: *Cocculina messingi*; Caenogastropoda: *Littorina littorea*). Our analysis incorporated previously published sequences plus new sequence data for *G. aegis* and *G chessoia*, using five standard markers: COI, histone 3 (H3), 16S rRNA, 18S rRNA and 28S rRNA*.* All five fragments were included for all taxa. COI sequences for the same individual specimens of *Gigantopelta* spp. were previously published as part of the species descriptions (GenBank accession numbers KR024336 for *G. chessoia*, KR024376 for *G. aegis*; [16]). New sequences generated from this study, for four fragments for each of the two *Gigantopelta* species are deposited in GenBank under accession numbers KX966246–KX966253.

The procedures for DNA extraction, amplification, purification, and sequencing are as detailed in Chen et al. [17]. The most suitable evolutionary model was tested using program PartitionFinder v. 1.0.1 [53], using scores for the Akaike information criterion. The models selected were as follows: H3, COI (first and second codons), 16S, 28S = GTR + I + G; COI (third codon) = HKY + I + G; 18S = K80 + G. The total concatenated sequence length used was 2753 bp.

Phylogenetic reconstruction was carried out with Bayesian inference using MrBayes v. 3.2 [54]. Metropolis-coupled Monte Carlo Markov Chains were run for five million generations. Convergence topologies were sampled every 100 generations and the first 25% were a priori discarded as burn-in after ensuring that chains sampled a stationary position. The software Tracer v. 1.6 [55] was used to check for convergence and to calculate adequate burn-in values.

### Anatomy

Observations from a combination of adult and juvenile specimens of *Gigantopelta* were compared with previous observations of other peltospirid taxa. One of the smallest juvenile specimens available for *Gigantopelta chessoia* (shell length ca. 3 mm) was selected for serial sectioning and the following 3D tomographic reconstruction. It was decalcified in 2% EDTA (pH 7.2) for 48 h, followed by subsequent dehydration in acetone series.

Prior to embedding, the specimen was stored in diluted Epon epoxy resin mixture (1:1 with 100% acetone) left uncovered overnight at room temperature, allowing acetone to evaporate. The specimen was then embedded in Epon with DPM-30 accelerator and hardened with the following protocol: 37 °C for 12 h, 45 °C for 12 h, and a further 24 h at 60 °C, according to the manufacturer’s instructions (Nissin EM, Japan). Samples were serially sectioned at a thickness of 1.5 μm using an ultramicrotome (Reichert Ultracut S; Leica, Wetzlar, Germany) fitted with a diamond knife (HistoJumbo 6 mm, DiATOME, Switzerland). Sections were stained using 0.1% toluidine blue and cover-slipped using Entellan New resin (Merk, Damstadt, Germany).

The serial semithin sections of the complete animal included 1332 sections; a subsample of every third section throughout the entire specimen was digitally captured using a camera unit mounted to a compound microscope trinocular (Olympus BX541), at an appropriate magnification to maximise specimen visibility. The resulting images were processed in Adobe Photoshop CC for contrast enhancement, size reduction, and converted to greyscale. The processed images were imported into Amira v5.3.3 (FEI Visualisation Sciences Group) and aligned into a single stack. In digital processing, materials of interest were highlighted throughout a subsampled image stack (444 section images) followed by post-processing included surface rendering and smoothing to generate the final tomographic model following previously published methods [45, 56, 57]. The slide-mounted serial sections are deposited in Zoologische Staatssammlung München (Munich, Germany) (ZSM Mol 20170157).

To generate a PDF file with the resulting full 3D anatomical model embedded, rendered surface (.surf) files were exported from Amira as.obj files and imported into DAZ Studio v4.9 (DAZ 3D). As colouration and transparency assignment were lost during export/import, identical values were copied from Amira and re-entered in DAZ Studio. After lighting and texture adjustments the full 3D model was exported as a single.u3d file. Adobe Acrobat XI Pro was used to import and embed this .u3d file in a blank PDF file. Finally, pre-set views were saved in Acrobat XI to complete the process.

### Electron microscopy

To investigate the shell microstructure, the shell of one adult specimen of *G. chessoia* and *G. aegis* each were broken from the aperture to produce shell pieces with fresh fractures. These were then observed uncoated with a Hitachi TM-3000 SEM.

To confirm the presence of intracellular microbes, a small sample from the central portion of the oesophageal gland of an adult *Gigantopelta chessoia* specimen was dehydrated in a graded acetone series and embedded in Epon resin (Sigma-Aldrich). Ultrathin sections (70 nm) were taken with an ultramicrotome (Reichert Ultracut S, Leica), and stained with 2% aqueous uranyl acetate and lead stain solution (0.3% lead nitrate and 0.3% lead acetate, Sigma-Aldrich). Transmission Electron Microscopy (TEM) was done at an acceleration voltage of 120 kV using a Tecnai 20 TEM (FEI).

## Additional file

Additional file 1: Interactive 3D Model. Description: 3D tomographic reconstruction of *Gigantopelta chessoia*, full interactive model. The interactive model can be enabled by clicking the figure (Adobe Acrobat Reader v7 or higher). Mouse *left click* and drag to rotate, hold down *ctrl* while doing so to move, and hold down *shift* while doing do to zoom (alternatively use mouse *right click* and drag or use the mouse wheel). Switch between pre-saved views using the dropdown menu in the floating window or click on the view pane in the model tree. Components can also be activated or deactivated by toggling the checkbox in the model tree. (PDF 7344 kb)
