# Supplementary material for: By more ways than one: Rapid convergence at hydrothermal vents shown by 3D anatomical reconstruction of Gigantopelta (Mollusca: Neomphalina)
Source: BMC Evol Biol. 2017 Mar 1;17:62. doi: 10.1186/s12862-017-0917-z (PMC5333402; doi:10.1186/s12862-017-0917-z)
Supplement: Additional file 1: — Interactive 3D Model. Description: 3D tomographic reconstruction of Gigantopelta chessoia, full interactive model. The interactive model can be enabled by clicking the figure (Adobe Acrobat Reader v7 or higher). Mouse left click and drag to rotate, hold down ctrl while doing so to move, and hold down shift while doing do to zoom (alternatively use mouse right click and drag or use the mouse wheel). Switch between pre-saved views using the dropdown menu in the floating window or click on the view pane in the model tree. Components can also be activated or deactivated by toggling the checkbox in the model tree. (PDF 7344 kb) [file 12862_2017_917_MOESM1_ESM.pdf]

Additional File 1: Interactive 3D Model

<u>Click on the image to activate the interactive model.</u>

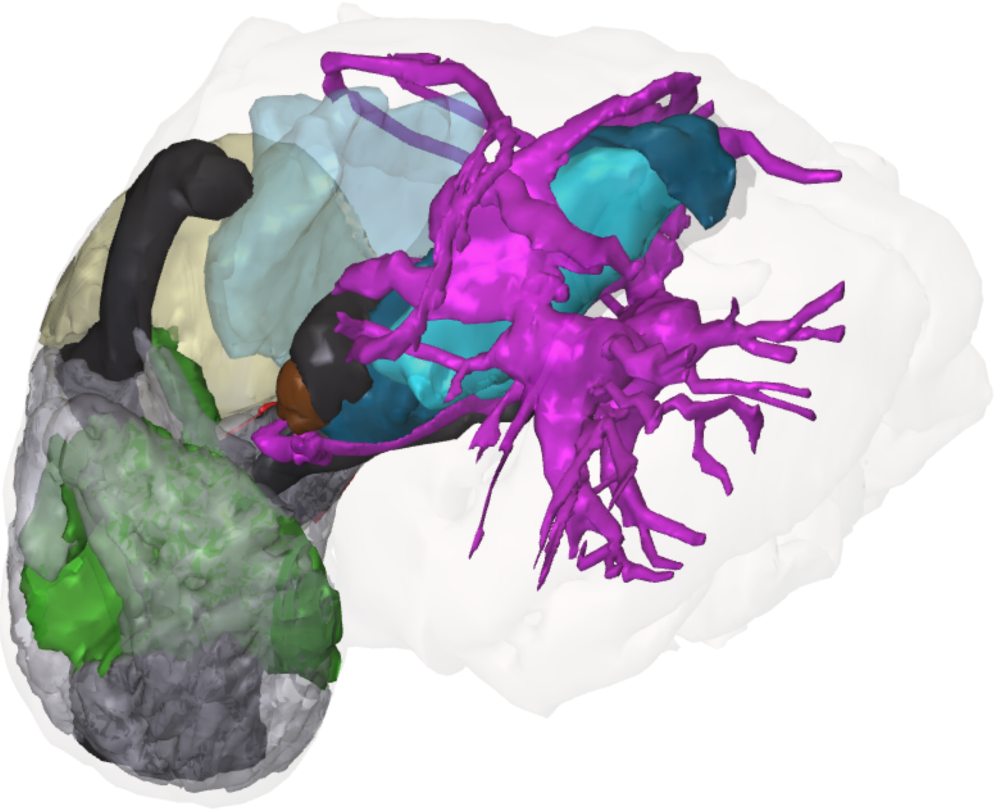

3D tomographic reconstruction of *Gigantopelta chessoia*, full interactive model. The interactive model can be enabled by clicking the figure (Adobe Acrobat Reader v7 or higher). Mouse *left click* and drag to rotate, hold down *ctrl* while doing so to move, and hold down *shift* while doing do to zoom (alternatively use mouse *right click* and drag or use the mouse wheel). Switch between pre-saved views using the dropdown menu in the floating window or click on the view pane in the model tree. Components can also be activated or deactivated by toggling the checkbox in the model tree.
